# Fourier transform in Bioinformatics and Biomedicine

**DOI:** 10.6026/973206300210575

**Published:** 2025-04-30

**Authors:** Paul Shapshak

**Affiliations:** 1 Department of Internal Medicine, Division of Infectious Diseases and International Health, Tampa General Hospital, Morsani College of Medicine, University of South Florida, Tampa, Florida 33606, USA

**Keywords:** Fourier transform (FT), bioinformatics, biomedicine, epidemiology, virology, genomics, sequences, DNA, RNA, protein, sequence analysis, alignments, phylogenetics, gene expression, protein interactions, networks, chromatin, simulation, molecular biology, signaling, biomedical signal processing, digital signal processing, wavelets, quantum computing

## Abstract

This brief paper provides an overview of the application of Fourier Transforms to bioinformatics and biomedicine. Fourier analysis and transforms have been applied extensively to fields including for example, cosmology, engineering and physics. In the fields of Bioinformatics and Biomedicine less has been accomplished. The application of Fourier analysis will amplify the accuracy and insights of these fields. Fourier analysis will also widen their scopes. This will improve understanding of basic fundamental biochemical and molecular processes and will assist in improving the accuracy and speed of medical and clinical diagnosis and treatments.

## Background:

Fourier Transforms (FTs) are mathematical techniques that convert functions of time or space into functions of frequency. In bioinformatics and biomedicine, FTs have been employed across various domains to analyze data, revealing patterns and structures that not easily discernible in the time or spatial domains [1]. This paper briefly discusses several applications of FT in bioinformatics and biomedicine.

## Multiple sequence alignment:

The study by Silverman and Linsker [2] utilized FT to accelerate the analysis of multiple sequences in large databases by converting sequences into frequency space. Their method efficiently identifies regions of similarity, making it a widely used tool in bioinformatics.

## DNA sequence codon analysis:

FT has been applied to identify coding regions in DNA sequences by detecting the three-base periodicity characteristic of amino acid codons. By transforming DNA sequences into numerical signals, FT can underscore these periodic patterns, aiding in gene prediction [3].

## Protein structure prediction:

Fourier transform infrared (FTIR) spectroscopy technique was employed to predict protein secondary structures by analyzing periodic patterns in amino acid sequences [4].

## Epidemiology:

In epidemiology, Wavelet analysis has been applied to study temporal patterns in disease incidence, helping to identify seasonal trends and periodic outbreaks. Measles infection spread from larger cities to smaller villages showed wave-like dynamics [5].

## Rapid protein alignment program MAFFT:

MAFFT rapidly produces multiple sequence alignments based on fast Fourier transform (FFT) method. This results in rapidly identified homologous regions [6].

## MAFFT applied to nucleic acids:

Modified MAFFT was developed to produce rapid alignments of nucleic acids, faster than Clustal [7].

## Virus disease:

Wavelet methodologies were developed and optimized to analyze the complex parameters and clinical and sub-clinical characteristics of hepatitis [8].

## DNA and protein sequence validity:

A molecular sequence information is digital and its rapid expansion continues at an accelerated rate producing huge volumes of data. Sequence information is transformed into character strings, which are then subject to digital signal processing (DSP). FT is used to identify features and properties - *e.g.* DNA and protein sequence validity can be assessed [9].

## Rapid time-resolved magnetic resonance angiography:

Rapid Time-Resolved Magnetic Resonance Angiography via a multi-echo radial trajectory and GraDeS reconstruction were used including FT to generate images of cerebral vasculature with distinction of venous and arterial separation [10].

## Quantum computing and FT:

In the realm of quantum computing and FT analyses, these have been applied to bioinformatics problems, such as aligning biological sequences. Quantum algorithms utilizing FT principles have been proposed to enhance computational efficiency in processing large-scale bioinformatics data [11, 12].

## Conclusion and prospects:

In conclusion, a great amount of work has been done utilizing Fourier analysis and transforms in physics, engineering and related fields [13]. However, comparatively less has been accomplished in Bioinformatics and Biomedical fields [1]. The scope of applications and analytic sophistication of Fourier analysis will strengthen the expanding fields of Bioinformatics and Biomedicine.
